# Novel cis-trans interactions are involved in post-transcriptional regulation of cyclin-dependent kinase inhibitor p21^WAF1/CIP1 ^mRNA

**DOI:** 10.1186/1750-2187-5-12

**Published:** 2010-08-12

**Authors:** Liyue Zhang, Anil Wali, Joseph A Fontana, Marcia I Dawson, Arun K Rishi

**Affiliations:** 1 Department of Internal Medicine, Wayne State University and John D. Dingell VA Medical Center, Room B4325, 4646 John R, Detroit, MI 48201, USA; 2 Department of Surgery and Karmanos Cancer Institute, Wayne State University, and John D. Dingell VA Medical Center, Room B4245, 4646 John R, Detroit, MI 48201, USA; 3 Department of Internal Medicine, Wayne State University and John D. Dingell VA Medical Center, Room B4324, 4646 John R, Detroit, MI 48201, USA; 4 Sanford-Burnham Medical Research Institute, La Jolla, CA 92037, USA; 5 Karmanos Cancer Institute, Department of Internal Medicine, Wayne State University and John D. Dingell VA Medical Center, Room B4325, 4646 John R, Detroit, MI 48201, USA; 6 Departments of Obstetrics & Gynecology, and Biochemistry University of Western Ontario Schulich School of Medicine and Dentistry, 4th Floor Victoria Research Labs, A4-130a 800 Commissioners Road East, ON N6C 2V5, London

## Abstract

**Background:**

A variety of pathways target CDKI p21^WAF1/CIP1 ^expression at transcriptional, post-transcriptional as well as translational levels. We previously found that cell growth suppressing retinoid CD437 enhanced expression of p21^WAF1/CIP1 ^and DNA damage inducible GADD45 proteins in part by elevating their mRNA stability.

**Results:**

Here, we investigated molecular mechanisms of CD437-dependent post-transcriptional regulation of p21^WAF1/CIP1 ^expression. By utilizing MDA-MB-468 HBC cells expressing chimeric rabbit β-globin-p21^WAF1/CIP1 ^transcripts we mapped multiple CD437-responsive sequences located within positions 1195 to 1795 of the 3'-untranslated region of p21^WAF1/CIP1 ^mRNA. Several cytoplasmic proteins present in MDA-MB-468, MCF-7 HBC as well as HL-60R leukemia cells bound specifically, in vitro, with these CD437-responsive sequences. CD437 treatment of cells resulted in elevated binding of ~85 kD and ~55 kD cytoplasmic proteins with putative CD437-responsive sequences. A 12 nt RNA sequence (5'-UGUGGUGGCACA-3') present within CD437-responsive region of p21^WAF1/CIP1 ^mRNA displayed specific and elevated binding with the above noted proteins. Treatment of cells with ActD or CHX prior to CD437 exposure did not abrogate RNA-protein interactions. However, treatment of cytoplasmic protein extracts with proteinase K or alkaline phosphatase resulted in loss of RNA-protein interactions.

**Conclusions:**

CD437 regulates cell growth in part by regulating stability of p21^WAF1/CIP1 ^mRNA that involves specific RNA-protein interactions that are phosphorylation-dependent, while not requiring nascent transcription or protein synthesis.

## Background

CDKI p21^WAF1/CIP1^, an important cell-cycle regulatory molecule, was originally identified as a gene regulated by the tumor suppressor protein p53 [1]. Subsequent studies also demonstrated induction of p21^WAF1/CIP1 ^via mechanisms independent of p53 [[2], refs within]. Indeed, exposure of cells to a wide variety of stress agents leads to induction of p21^WAF1/CIP1^, which, in turn, participates in mediating cell-cycle arrest. It is thought that cell-cycle arrest due to induction of p21^WAF1/CIP1 ^following exposure of cells to stress stimuli, functions as a protective factor in determining the survival of the cell. The mechanisms underlying stress-induced expression of p21^WAF1/CIP1 ^have been found to involve both transcriptional as well as post-transcriptional processes [[2], refs within]. Numerous transcription factors, including p53, Sp1, p300, CEBPβ, AP2, and STATs, have previously been shown to regulate p21^WAF1/CIP1 ^transcription [[2], refs within]. In addition, the transcription factor CEBPα has been found to associate with the p21^WAF1/CIP1 ^protein and as a result considerably extended the p21^WAF1/CIP1 ^protein half-life [3]. Regulation of mRNA turnover has also been found to be an important determinant in the stress-induced expression of p21^WAF1/CIP1 ^[4-7].

CD437 belongs to a class of adamantyl-substituted retinoids that suppress growth by inducing cell-cycle arrest as well as apoptosis in a number of cell types including HBC, prostate carcinoma, leukemia and normal mammary epithelial cells [8-11]. CD437, designed as an retinoic acid receptor (RAR)γ-selective molecule, exerts its action through RAR/retinoid X nuclear receptor (RXR)-independent as well as p53-independent pathway. CD437 treatment induces expression of p21^WAF1/CIP1 ^mRNA and protein in a number of cell types [8-11]. In this report we demonstrate that CD437-dependent induction of p21^WAF1/CIP1 ^in HBC cells is accomplished, in part, by targeting multiple, novel sequences within an approximately 600 nt subfragment of the 3'-UTR of p21^WAF1/CIP1 ^mRNA. Further, we report identification of a 12 nt p21^WAF1/CIP1 ^mRNA sequence that shows specific binding, in vitro, with multiple cytoplasmic protein complexes present in HBC and HL-60R leukemia cells. CD437 causes elevated binding, in vitro, of ~55 kD and 85 kD-sized cytoplasmic complexes with these sequences.

## Results

### The 3'-UTR of p21^WAF1/CIP1 ^harbors destabilizing and CD437-responsive cis element(s)

**Figure 1:**
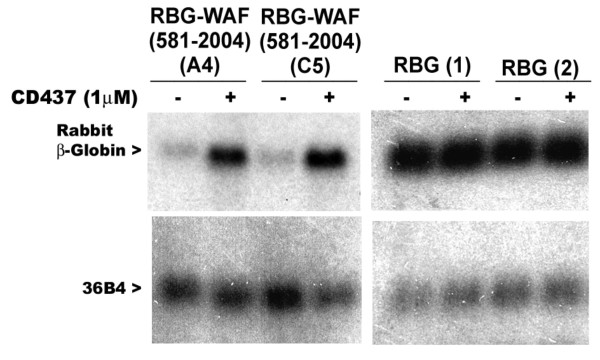
**CD437 regulation of chimeric rabbit β-globin-p21^WAF1/CIP1 ^mRNA**. Two independent sublines derived from transfection of RGB-WAF (581-2004) plasmid [31.4(A4) and 31.4(C5)] or RBG plasmid [29.6(1) and 29.6(2)] were grown either in the absence (-) or presence (+) of 1 μM CD437 for 48 hours. Total RNAs were prepared and expression of RBG transcripts analyzed by northern blot hybridization as described [12]. Levels of RNA loading in each lane were assessed by signals from hybridization with ribosomal phosphoprotein 36B4 cDNA [18]

**Figure 2:**
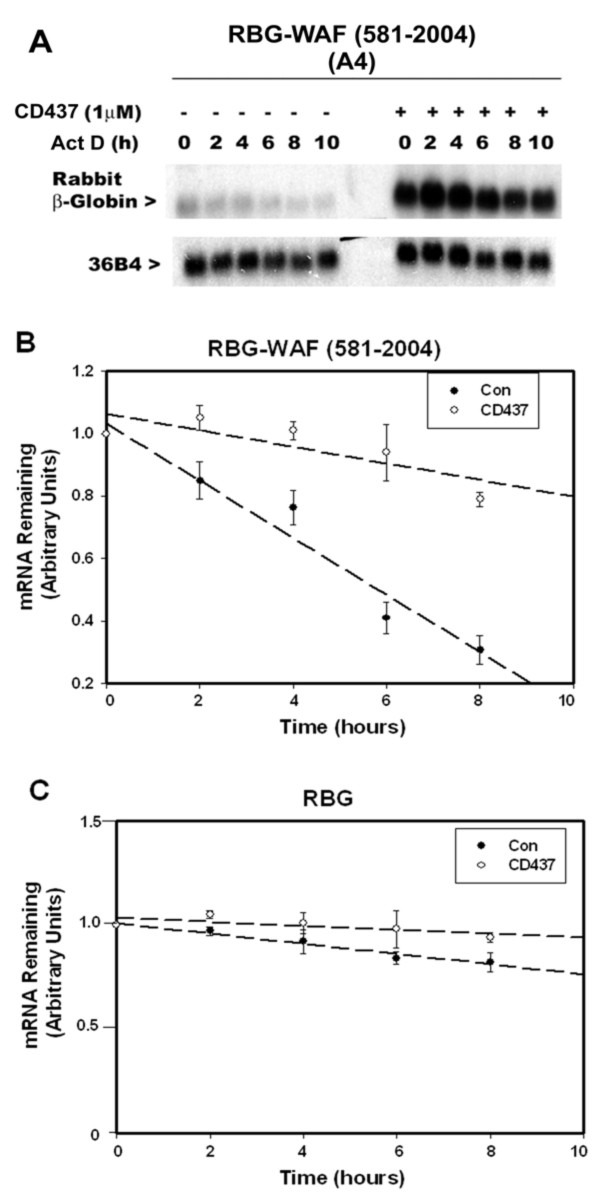
**Presence of p21^WAF1/CIP1 ^3'-UTR causes destabilization of RBG mRNA, while CD437 treatments enhance stability of chimeric RBG-WAF transcripts**. MDA-MB-468 sublines derived from stable transfection of RBG-WAF (581-2004) plasmid (Panel **A**) or RBG plasmid (not shown) were cultured in the absence (-) or presence (+) of CD437 for 48 hours and exposed to transcriptional inhibitor ACT D for noted times. The preparation of RNA and northern blot hybridization were as described in the legend to figure 1. Panels **B **and **C **show plots of the decay of RBG transcripts in either the presence or absence of CD437. The RBG mRNA levels at time 0 in either the absence or presence of CD437 were arbitrarily defined as 1. Data in panels B and C represent mean of four and two independent sublines, respectively, for each time point. Error bars represent the standard error of the mean.

To determine whether sequences present in the p21^WAF1/CIP1 ^3'-UTR cause instability of heterologous rabbit βglobin (RBG) mRNA, as well as harbor CD437-responsive elements, we utilized MDA-MB-468 HBC sublines that stably express RBG or RBG-WAF (581-2004) transcripts in the following experiments. First, the cells were either untreated or treated with 1 μM CD437 followed by analysis of expression of RBG or RBG-WAF transcripts by northern blot hybridization as described in the methods. CD437 treatment resulted in elevated levels of RBG-WAF (581-2004) transcripts, while no such increase in expression of RBG mRNA was noted (figure 1). Next, multiple, independent sublines expressing RBG or RBG-WAF (581-2004) transcripts were separately incubated in the presence or absence of CD437 for 40 hours, followed by their treatments with 4 μg/ml ActD for additional 2, 4, 6, 8, or 10 hours. Total cellular RNAs were isolated and expression of RBG and RBG-WAF (581-2004) transcripts analyzed by northern blot as in Methods. As shown in figure 2A, CD437 treatments enhanced expression of chimeric RBG-WAF (581-2004) transcripts. The expression of RBG mRNA, on the other hand, was unaltered following similar treatments with ActD in the absence or presence of CD437 (data not shown). Next, the northern blot data from two and four independent sublines expressing RBG and RBG-WAF (581-2004) mRNAs, respectively, that were treated with ActD in the absence or presence of CD437 as in figure 2A, was utilized in calculating rate of decay of the RBG and RBG-WAF (581-2004) transcripts. The half-life (t_½_) of RBG and RBG-WAF (581-2004) transcripts was calculated as described before [12]. The RBG-WAF (581-2004) transcripts displayed a half-life of ~4 h in comparison to RBG transcripts that were found to be very stable (figures 2B** and **2C). Thus, the data in figure 2 suggest that the 3'-UTR of p21^WAF1/CIP1 ^contains sequences that affect stability of the RBG transcripts in the HBC cells. Further, CD437 treatment of HBC cells expressing RBG-WAF (581-2004) transcripts resulted in significant enhancement (~4-fold) of the stability of the chimeric transcripts (figure 2B). Together, data in figures 1 and 2 demonstrate that the ~1.4 kb long 3'-UTR of p21^WAF1/CIP1 ^mRNA contains elements that regulate stability of RBG mRNAs in HBC cells dependent as well as independent of CD437.

### Multiple CD437-responsive sequences exist within p21^WAF1/CIP1 ^3'-UTR

**Figure 3:**
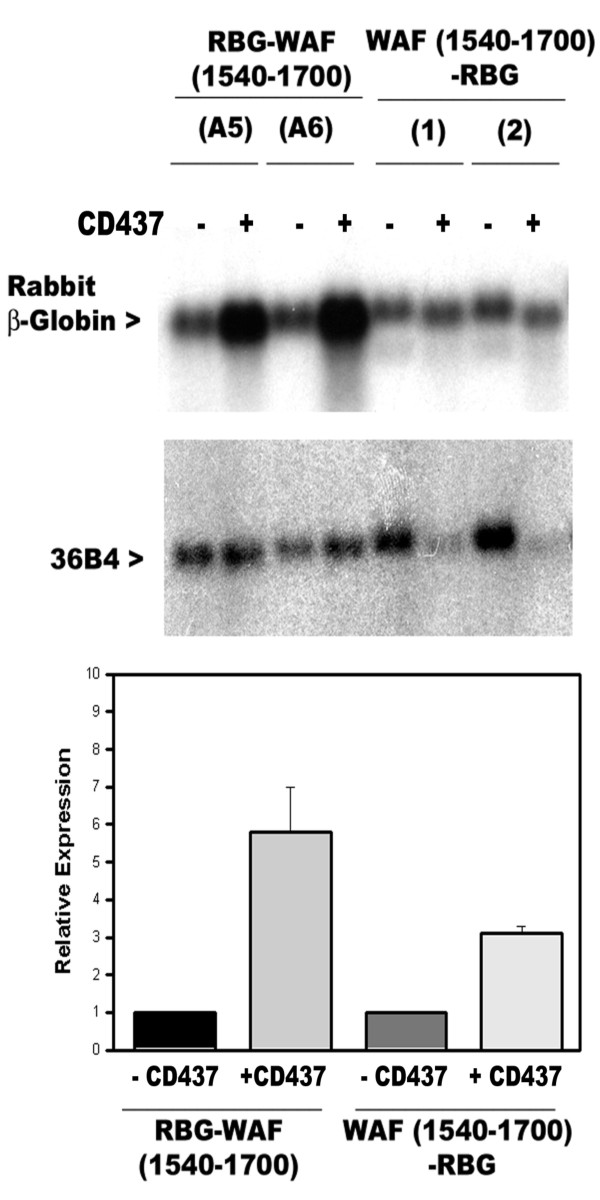
**CD437 targets 3'-UTR sequences of p21^WAF1/CIP1 ^mRNA independent of their location in the chimeric RBG transcripts**. **(A) **Two independent sublines derived from stable transfection of each of the RBG-WAF (1540-1700) plasmid (labelled as A5 and A6) and WAF (1540-1700)-RBG plasmid (labeled as 1, 2) were grown either in the absence (-) or presence (+) of 1 μM CD437 for 48 hours. The cellular RNAs were prepared and northern blot hybridizations carried out as described in the legend to figure 1. **(B) **Histogram showing relative expression of chimeric RBG-WAF transcripts in control vs CD437-treated sublines in panel A. Northern blots of panel A were quantitated by soft laser densitometry. Columns represent mRNA levels relative to the levels of mRNAs present in the untreated control lanes, which were arbitrarily defined as 1. The error bars represent the standard error of the mean.

To further map CD437-responsive sequences, independent sublines harboring plasmids expressing various chimeric RBG-WAF transcripts were utilized (table 1). Each of the sublines was independently cultured in the presence or absence of CD437 (1 μM) for 48 hours, followed by extraction of total RNA and northern blot hybridization to determine expression of chimeric transcripts (not shown). As summarized in table 1, CD437 treatments resulted in elevated expression of RBG-WAF transcripts in all the sublines except those expressing RBG-WAF (1795-2004), RBG-WAF (1900-2004), RBG-WAF (581-1195), and RBG-WAF (581-1011) transcripts indicating the presence of CD437-responsive cis elements within positions 1195 to 1795 of p21^WAF1/CIP1 ^mRNA [1]. As also noted in table 1, CD437 stimulated expression of chimeric transcripts in HBC sublines harboring RBG-WAF (1645-2004), RBG-WAF (1498-1645), and RBG-WAF (581-1498) constructs that have non-overlapping fragments of p21^WAF1/CIP1 ^3'-UTR. These data support the possibility of multiple, independently functional CD437-responsive sequences within the p21^WAF1/CIP1 ^3'-UTR. Interestingly, CD437 treatment of HBC sublines expressing the WAF (1540-1700)-RBG transcript (table 1), in which the p21^WAF1/CIP1 ^mRNA sequences were positioned at the 5' end of the RBG transcript also resulted in elevated levels of chimeric transcripts (figure 3). Since, the plasmids RBG-WAF (1540-1700) and WAF (1540-1700)-RBG have sense orientation of identical p21^WAF1/CIP1 ^3'-UTR sequences (from positions 1540-1700, [1]) positioned at the 3' and 5' ends, respectively, of the RBG gene, the elevated expression of chimeric transcripts derived from these constructs noted in figure 3 further underscore the fact that CD437-responsive RNA sequences function independent of their location in the target mRNA. The data in figure 3 thus corroborate our previous finding where CD437-dependent expression of GADD45 involved RNA sequences located at the 5'-UTR of GADD45 mRNA [12].

**Table 1:** CD437-responsive sequences of p21^WAF1/CIP1 ^3'-UTR

Construct ID	Plasmid Name	**Positions of p21**^**WAF1/CIP1 **^**3'-UTR insert **[4]	CD437 Responsiveness
29.6	RBG	-------	-------

31.4	RBG-WAF (581-2004)	+581 to +2004	Yes

30.2	RBG-WAF (1006-2004)	+1006 to +2004	Yes

32.8	RBG-WAF (1498-2004)	+1498 to +2004	Yes

33.7	RBG-WAF (1645-2004)	+1645 to +2004	Yes

36.4	RBG-WAF (1795-2004)	+1795 to +2004	No

37.4	RBG-WAF (1900-2004)	+1900 to +2004	No

38.1	RBG-WAF (1498-1645)	+1498 to +1645	Yes

49.1	RBG-WAF (1540-1700)	+1540 to +1700	Yes

50.1	WAF (1540-1700)-RBG	+1540 to +1700	Yes

60.3	RBG-WAF (581-1498)	+581 to +1498	Yes

61.2	RBG-WAF (581-1195)	+581 to +1195	No

62.3	RBG-WAF (581-1011)	+581 to +1011	No

### Multiple cytoplasmic proteins bind specifically to CD437-responsive UTR subfragments of p21^WAF1/CIP1 ^and GADD45 mRNAs, and their binding is enhanced following treatment of cells with CD437

**Figure 4:**
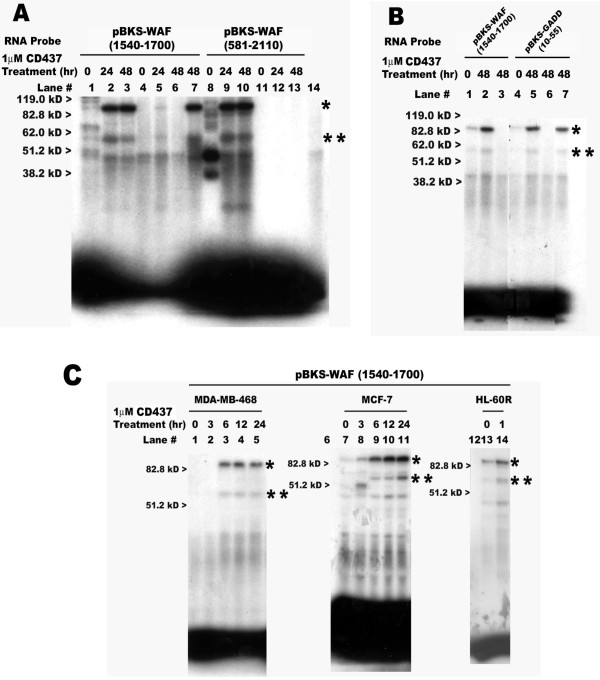
**Multiple cell cytoplasmic proteins bind to radiolabelled p21^WAF1/CIP1 ^3'-UTR and GADD45 5'-UTR sense strand riboprobes**. Transcription and labeling of the indicated probes as well as the in vitro binding reactions were as described in the Methods. The probes utilized and treatment times with CD437 (hr) are indicated above each lane in the respective panels. The cytoplasmic protein extracts were prepared from untreated (lanes marked 0 hours) or CD437-treated MDA-MB-468 (panels A-C), MCF-7 (panel C) and HL-60R (panel C) cells. **Panel A, **the protein extracts in lanes 4-6 and11-13 were pre-incubated with 200-fold excess of the respective unlabeled probe RNAs, while the protein extracts in lane 7 were pre-incubated with 200-fold excess of the unlabeled 3'-UTR of c-myc RNA followed by incubation with the indicated labeled probe RNAs. Probe 14.2 RNA is loaded in lane 14. **Panel B**, the protein extracts in lanes 3 and 6 were pre-incubated with 200-fold excess of the respective unlabeled probe RNAs, while the protein extracts in lane 7 were pre-incubated with 200-fold excess of the unlabeled 3'-UTR of c-myc RNA followed by incubation with the indicated labeled probe RNAs. **Panel C**, lanes 6, 12 represent the labeled probe only. The approximate migration of molecular weight standards is marked on the left side of respective panel, while the * and ** on the right side of each panel denote locations of the putative 85 kD and 55 kD complexes, respectively.

The possibility of specific RNA-protein interactions involved in CD437-dependent regulation of p21^WAF1/CIP1 ^and GADD45 expression was investigated as follows. First, pBKS-WAF (581-2110), pBKS-WAF (1540-1700), and pBKS-GADD (10-55) plasmids (table 2) were utilized to synthesize unlabelled as well as radiolabelled RNAs as described in Methods. Next, the in vitro binding of the cellular cytoplasmic proteins derived from untreated as well as CD437-treated HBC cells to the above radiolabelled probes was investigated by UV-induced cross-linking. The 160 nt subfragment of p21^WAF1/CIP1 ^3'-UTR present in the plasmid pBKS-WAF (1540-1700) binds specifically with multiple cytoplasmic protein complexes, and pre-treatment of HBC cells with CD437 results in elevated binding with three major cytoplasmic protein complexes of ~85, 55, and 40 kD sizes (figure 4; panel A, lanes 2, 3; panel B, lane 2). The presence of 200-fold excess of this unlabelled probe abolished the above RNA-protein interactions (figure 4; panel A, lanes 4, 5, 6; panel B, lane 3), while these interactions were unaffected by the presence of 200-fold excess of non-specific RNA (figure 4; panel A, lane 7). Like the p21^WAF1/CIP1 ^3'-UTR subfragment probe, the GADD45 5'-UTR probe derived from pBKS-GADD (10-55) plasmid (table 2) also showed specific binding with multiple cytoplasmic complexes and pre-treatment of HBC cells with CD437 caused elevated binding of the above noted three major cytoplasmic protein complexes of ~85, 55, and 40 kD sizes (Figure 4B, lane 5). The binding of these complexes was also abolished by a 200-fold excess of unlabelled probe (figure 4B, lane 6), but remained unaffected by the presence of 200-fold excess of non-specific RNA (figure 4B, lane 7). Furthermore, labeled probe synthesized from plasmid pBKS-WAF (581-2110) also showed specific binding to several cytoplasmic protein complexes derived from untreated as well as CD437-treated HBC cells (figure 4A). In addition to the above noted ~85, 55 and 40 kD complexes, several other RNA-protein complexes were found in the lane containing extracts from untreated HBC cells (lane 8, figure 4A). The elevated and specific binding of several RNA-protein complexes, including 85, 55, and 40 kD, as well as additional ~25 and 35 kD species was evident in the lanes having extracts derived from CD437-treated HBC cells (figure 4A; lanes 9, 10). Together, data in figure 4A and 4B, suggest that CD437 causes enhanced expression of p21^WAF1/CIP1 ^and GADD45 mRNAs in HBC cells, in part, by targeting specific RNA-protein interactions. These RNA-protein interactions involve elevated binding of putative 85, 55, and 40 kD proteins to the CD437-responsive target RNA sequences.

**Table 2:** Plasmid constructs for *in vitro *transcription

Construct I.D.	Plasmid Name	Positions of the UTR inserts	Gene [ref]
WAF 14.2	pBKS-WAF (518-2110)	3'-UTR +581 to +2110	P21^WAF1/CIP1 ^[1]

WAF 52.1	pBKS-WAF (1540-1710)	3'-UTR +1540 to +1700	P21^WAF1/CIP1 ^[1]

WAF 55.1	pBKS-WAF (1540-1585)	3'-UTR +1540 to +1585	P21^WAF1/CIP1 ^[1]

WAF 56.5	pBKS-WAF (1575-1620)	3'-UTR +1575 to +1620	P21^WAF1/CIP1 ^[1]

WAF 57.8	pBKS-WAF (1610-1655)	3'-UTR +1610 to +1655	P21^WAF1/CIP1 ^[1]

WAF 58.3	pBKS-WAF (1645-1690)	3'-UTR +1645 to +1690	P21^WAF1/CIP1 ^[1]

GADD 12.4	pBKS-GADD (10-55)	5'-UTR +10 to +55	GADD45 [13]

### Cytoplasmic proteins interacting with CD437-responsive p21^WAF1/CIP1 ^3'-UTR subfragment are also present in different cell types

To further elucidate RNA cis-trans interactions involved in post-transcriptional regulation of p21^WAF1/CIP1 ^expression, cytoplasmic extracts from untreated and CD437-treated MDA-MB-468 and MCF-7 HBC cells as well as HL-60R human leukemia cells were prepared as described in Methods. Radio-labeled probe derived from pBKS-WAF (1540-1700) plasmid (table 2) was utilized to study *in vitro *binding of cytoplasmic proteins. Figure 4C shows that pretreatment of HBC as well as leukemia cells with CD437 results in elevated binding of the above noted ~85 kD, 55 kD and 40 kD cytoplasmic protein complexes (lanes 3-5, 8-11, and 14). The elevated RNA-protein interactions were noted following 3-6 hours and 1 hour treatments of HBC (MCF-7 and MDA-MB-468) and leukemia cells, respectively. The data in figure 4C thus are consistent with earlier observations where 1 and 6 hours of CD437 treatments caused induction of p21^WAF1/CIP1 ^in HL-60R leukemia and HBC cells, respectively [8,12]. Together, the data in figure 4 underscore the conservation of RNA-protein interactions involved in the regulation of p21^WAF1/CIP1 ^and GADD45 expression in different cell types in the presence of CD437.

### RNA cis-trans interactions with the CD437-responsive p21^WAF1/CIP1 ^3'-UTR subfragment are phosphorylation-dependent

**Figure 5:**
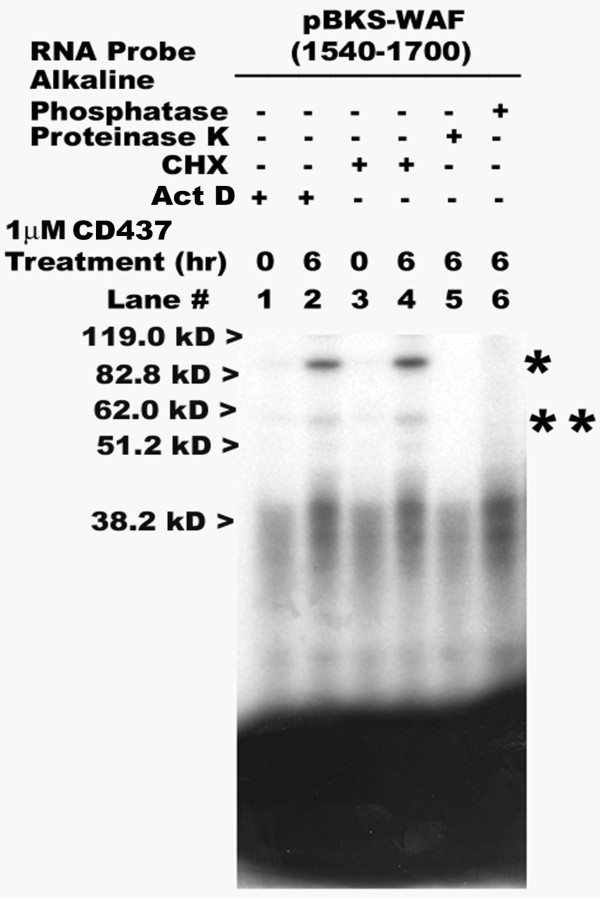
**CD437-dependent RNA-protein interactions are independent of nascent protein synthesis or transcription but are phosphorylation dependent**. The preparation of cytoplasmic protein extracts from untreated (lanes 0 hour) or CD437-treated (lanes 2, and 4-6, 6 hours) MDA-MB-468 HBC cells, and transcription and labeling of the indicated probe as well as in vitro binding reactions were as described in the methods. The cytoplasmic extracts were prepared from HBC cells pre-treated with protein synthesis inhibitor CHX (lanes 1, 2) or transcriptional inhibitor ActD (lanes 3, 4) followed by treatments with CD437 (time and dose indicated). In addition, cytoplasmic protein extracts derived from CD437-treated HBC cells were pretreated with alkaline phosphatase (lane 6) or proteinase K (lane 5) prior to binding with the indicated probe. The approximate migration of molecular weight standards is marked on the left side, whereas the * and ** on the right side of the panel denote locations of the putative 85 kD and 55 kD complexes, respectively.

To investigate the molecular basis of RNA-protein interactions targeted by CD437, the following experiments were conducted. MDA-MB-468 HBC cells were pretreated with either transcriptional inhibitor ActD or protein synthesis inhibitor CHX for 1 hour, followed by their culture in the absence or presence of 1 μM CD437 for an additional 6 hours. The cytoplasmic extracts were prepared and RNA-protein interactions analyzed as above. In addition, HBC cell cytoplasmic extracts that were prepared after 24 hours of 1 μM CD437 treatment were subjected to either alkaline phosphatase or Proteinase K as detailed in Methods prior to their utilization in binding reactions *in vitro*. figure 5, lanes 2 and 4, show that pretreatment with either ActD or CHX failed to abolish the CD437-dependent interactions of the above noted ~85 kD, 55 kD, and 40 kD complexes indicating that these CD437-dependent RNA-protein interactions are independent of nascent transcription as well as protein synthesis. However, the pretreatment of cytoplasmic protein extracts with Proteinase K resulted in loss of the binding of the 85 kD and 55 kD complexes while the binding of the ~40 kD and smaller size complexes was significantly reduced (figure 5, lane 5). In addition, the pretreatment of cytoplasmic protein extracts with alkaline phosphatase resulted in loss of binding of the 85 kD and 55 kD complexes but did not affect the binding of the ~40 kD and smaller size complexes (figure 5, lane 6). Thus, the data in figure 5 suggest that the CD437-dependent interactions of ~85 kD and 55 kD proteins, but not the 40 kD and smaller-sized complexes with the p21^WAF1/CIP1 ^3'-UTR subfragment are dependent on phosphorylation.

### A 12 nt sequence of the p21^WAF1/CIP1 ^3'-UTR has a CD437-responsive cis-element

**Figure 6:**
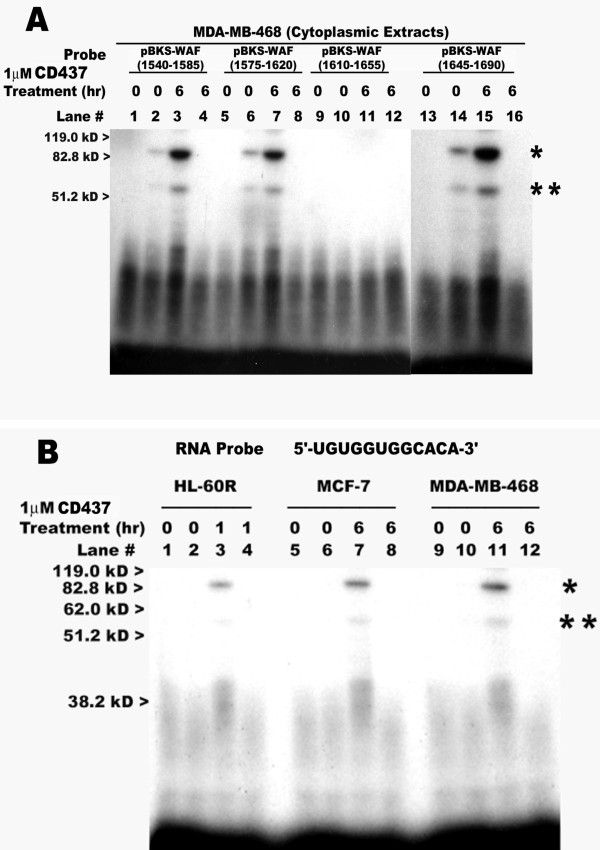
**Multiple CD437-responsive sequence elements exist within the 3'-UTR of p21^WAF1/CIP1^**. Transcription and labeling of the indicated probes and binding reactions were as described in the Methods. The probes utilized and treatment times with CD437 (hours, hr) are indicated in the respective panels. In panel B, the probe sequence of the 12 nt RNA oligonucleotide is shown. The cytoplasmic protein extracts were prepared from untreated (lanes marked 0) or CD437-treated MDA-MB-468 (panels A, B), MCF-7 (panel B) and HL-60R (panel B) cells. The respective probes only were loaded in lanes 1, 5, 9, and 13. The protein extracts in lanes 4, 8, 12, and 16 were pre-incubated with 200-fold excess of the unlabeled RNA derived from pBKS-WAF (1540-1700) plasmid (table 2), followed by incubation with the indicated labeled probe. The approximate migration of molecular weight standards is marked on the left side of respective panel, while the * and ** on the right side of each panel denote locations of the putative 85 kD and 55 kD complexes, respectively.

To map CD437-responsive sequences present within the above 160 nt 3'-UTR RNA subfragment (positions 1540 to 1700, [1]), additional smaller sized fragments were created by synthesizing oligonucleotides and subsequently cloning the 48 nt long inserts into vector plasmid pBKSII as detailed in Methods. The resultant pBKS-WAF (1540-1585), pBKS-WAF (1575-1620), pBKS-WAF (1610-1655) and pBKS-WAF (1645-1690) plasmids (table 2) were then independently linearized with XbaI, followed by synthesis of radiolabelled sense strand RNAs. As observed in the case of the RNA probe derived from the pBKS-WAF (1540-1700) plasmid (figure 4B), all of the 45 nt overlapping RNA probes, with the exception of that derived from the pBKS-WAF (1610-1655) plasmid, showed specific binding with the putative ~85 kD and ~55 kD complexes (figure 6A, lanes 2, 3, 6, 7, 14, and 15). The binding of the proteins to the RNA probes derived from the pBKS-WAF (1540-1585), pBKS-WAF (1575-1620) and pBKS-WAF (1645-1690) plasmids was elevated in the case of cytoplasmic extracts obtained from HBC cells treated with CD437 for 6 hours (figure 6A, lanes 3, 7, and 15). Thus, the similarity of the RNA-protein interactions observed in figure 6A in the case of non-overlapping 45 nt RNA probe fragments derived from pBKS-WAF (1540-1585) and pBKS-WAF (1645-1690) plasmids suggests the presence of multiple CD437-responsive sequences within positions 1540-1700 of p21^WAF1/CIP1 ^mRNA. This observation further supports the data presented in table 1, where HBC sublines expressing RBG-WAF (581-1498), RBG-WAF (1498-1645) and RBG-WAF (1645-2004) transcripts that contain non-overlapping subfragments of the p21^WAF1/CIP1 ^3'-UTR displayed CD437-dependent increased expression of chimeric transcripts. Taken together, the data in figures 4C, 6A and table 1 strongly support existence of multiple CD437-responsive cis-sequences that specifically interact with ~55 kD and ~85 kD cytoplasmic protein complexes to elicit CD437-dependent elevated expression of p21^WAF1/CIP1 ^in HBC and leukemia cells.

As observed in figure 6A, the 45 nt overlapping RNA probes derived from pBKS-WAF (1540-1585) and pBKS-WAF (1575-1620) plasmids interacted independently with the similar sized HBC cytoplasmic proteins. To map the CD437-responsive cis element, a 12 nt RNA oligonucleotide from positions 1574-1585 was synthesized and subsequently utilized in RNA-protein interactions *in vitro *in conjunction with the cytoplasmic extracts derived from untreated or CD437-treated HBC (MCF-7 and MDA-MB-468) as well as HL-60R leukemia cells as above. As expected, the elevated and specific binding of ~85 kD, 55 kD, 40 kD and smaller sized complexes with the 12 nt probe was observed in the cytoplasmic extracts obtained from CD437-treated leukemia as well as HBC cells (figure 6B, lanes 3, 7, and 11).

## Discussion

Expression of the CDKI p21^WAF1/CIP1 ^during the stress response is regulated at multiple levels including transcriptional, post-transcriptional and post-translational [[2] and refs within]. Expression of p21^WAF1/CIP1 ^in response to stress stimuli utilizes both the tumor suppressor p53-dependent as well as p53-independent pathways. During cell-cycle progression, p21^WAF1/CIP1 ^expression fluctuates with peaks at the G_1 _and G_2 _phases of the cell cycle. Accumulating evidence suggests that stress-induced expression of p21^WAF1/CIP1^, in general, protects against stress in order to enhance cell survival by temporarily suppressing cell growth. We have recently observed that exposure of different cell types, including HBC, prostate, leukemia and normal mammary epithelial cells to several adamantyl-substituted retinoids results in rapid induction of p21^WAF1/CIP1 ^followed by apoptosis [8-11]. CD437-dependent induction of p21^WAF1/CIP1 ^in HBC cells was shown to utilize both the transcriptional and post-transcriptional mechanisms independent of cellular p53 [13]. CD437-dependent regulation of p21^WAF1/CIP1 ^in the HBC cells involved, in part, enhanced message stability utilizing cis sequences present in the 3'-UTR [13].

In this report, we conducted experiments to further characterize the CD437-responsive cis sequences present in the 3'-UTR of p21^WAF1/CIP1 ^mRNA. Using deletion analyses in combination with *in vitro *RNA-protein binding approaches, we found existence of multiple novel CD437-responsive cis elements and their binding to specific, perhaps novel, trans-acting cytoplasmic proteins. We identified a 12 nt RNA sequence (5'-UGUGGUGGCACA-3', figure 6) that showed elevated binding to specific cytoplasmic proteins present in the extracts derived from CD437-treated HBC and leukemia cells. We also found that CD437 treatment of HBC cells caused elevated expression of chimeric RBG-WAF transcripts that contain CD437-responsive sequences of p21^WAF1/CIP1 ^mRNA. Since the above 12 nt sequence is part of the CD437-responsive p21^WAF1/CIP1 ^mRNA region, the data in this report strongly implicate the observed RNA-protein interactions in CD437-dependent regulation of p21^WAF1/CIP1 ^expression. It should, however, be noted that the p21^WAF1/CIP1 ^3'-UTR RNA sequence of pBKS-WAF (1645-1690) by not overlapping with above 12 nt sequence yet binding with protein complexes that were similar to those observed in the case of 12 nt sequence (see figure 6A, table 2) highlights the possibility of degeneracy among the multiple, CD437-responsive sequences present in the 3'-UTR of p21^WAF1/CIP1 ^mRNA.

Previously, Elav-like RNA-binding proteins were demonstrated to bind, *in vitro*, to a 42 nt U-rich sequence present in the 3'-UTR of p21^WAF1/CIP1 ^mRNA [5]. HuR, a HuD-related protein of the Elav-like RNA-binding protein family was indeed found to regulate p21^WAF1/CIP1 ^mRNA stabilization induced by UV light in human RKO colorectal carcinoma cells [7]. That the above 12 nt sequence is a novel motif utilized by CD437 to regulate p21^WAF1/CIP1 ^mRNA expression is borne out by the following observations. First, the 12 nt sequence identified in this report is located within a 3'-UTR region that is different from the previously identified 3'-UTR subfragment implicated in binding to the Elav-like mRNA stabilizing proteins [5]. Second, transient transfection of a plasmid expressing the CMV promoter-driven luciferase reporter gene containing the AU-rich region subfragment of p21^WAF1/CIP1 ^mRNA into HBC cells failed to elicit CD437-dependent enhanced luciferase activity [13]. Third, the Elav-like proteins that bind the p21^WAF1/CIP1 ^mRNA 3'-UTR consist, in general, of proteins with a molecular weight range of 35-50 kD [5,7]. The CD437-dependent RNA-protein interactions observed in this report consist of multiple complexes that include 85 kD, 55 kD, 40 kD and smaller-sized complexes that interact with the above 12 nt sequence. It remains to be determined whether the CD437-dependent elevated binding of 40 kD and smaller-sized complexes with the 12 nt sequence indeed involve any Elav-like RNA binding proteins. Fourth, the essentially similar nature of RNA-protein interactions was observed when different RNA probes derived from p21^WAF1/CIP1 ^and GADD45 mRNAs [pBKS-WAF (1540-1700) and pBKS-GADD (10-55); figure 4B] as well as the above 12 nt sequence were separately utilized in conjunction with the cytoplasmic extracts derived from CD437-treated HBC cells (see figures 4 and 6). The 45 nt probe derived from the 5'-UTR of GADD45 mRNA [pBKS-GADD (10-55, [12]) has high GC content and lacks previously characterized AU-rich elements. Its presence at the 5' end of the heterologous rabbit β-globin gene caused enhanced expression of chimeric β-globin transcripts in CD437-treated HBC cells [12,14]. Taken together, the CD437-mediated RNA-protein interactions described in this report suggest the existence of novel pathway(s) targeting the steady-state levels of cellular mRNAs independent of the involvement of the AU-rich regions of the target mRNAs.

Several labile cellular RNAs are regulated by pathways that control their degradation. The pathways regulating steady state levels of cellular RNAs involve interactions of a number of proteins with cis sequences located in the UTRs of the target RNAs [15]. The most common mRNA stability elements involve AU-rich sequences present in the 3'-UTRs of several short-lived mRNAs including those encoding for cytokines (interleukins and interferons), oncogenes (c-myc, c-fos and c-Jun), and genes regulating cell cycle (for example, p21^WAF1/CIP1^). The RNA-protein interactions identified in this study being independent of the pathways involving AU-rich sequences is supported by experiments in which the unlabelled c-myc 3'-UTR (figures 6) or p21^WAF1/CIP1 ^3'-UTR AU-rich region RNA (clone 7.1, [13], data not shown) sequences failed to compete for the binding of the cytoplasmic proteins with radiolabelled probes containing CD437-responsive p21^WAF1/CIP1 ^sequences. Taken together, the data suggest that the HBC proteins interacting with the CD437-responsive cis sequences are different, perhaps novel, proteins than those binding with the AU-rich region subfragments of c-myc and p21^WAF1/CIP1 ^RNAs.

It has been suggested that the factors binding with the AU-rich RNA elements involved post-translational modification such as phosphorylation [16,17]. Indeed, as shown in figure 5, pre-treatment of HBC cell cytoplasmic extracts with alkaline phosphatase abolished the interactions of the proteins with the CD437-responsive sequences. Although, CD437 did not induce p21^WAF1/CIP1 ^expression in all cell-types, the specific phosphorylation-dependent binding of above 85 kD and 55 kD proteins to a novel RNA cis sequence may constitute an important pathway regulating the steady-state levels of CD437 target genes in HBC as well as leukemia cells. Whether factors binding to AU-rich RNA elements and CD437-responsive sequences share upstream kinase(s) and/or phosphatase(s) that may regulate cis-trans interactions remains to be elucidated.

## Conclusions

Novel cis sequences located within the 3'-UTR of CDKI p21^WAF1/CIP1 ^regulate its expression and consequent cell growth in the presence of the apoptosis-promoting adamantyl-substituted retinoid CD437. CD437 induces specific, phosphorylation-dependent binding of multiple cytoplasmic proteins with the p21^WAF1/CIP1 ^mRNA 3'-UTR sequences in a manner independent of the cellular p53 and retinoid receptor status [8,13].

## Methods

### Materials

DMEM, Ham's F-12 medium, and FBS were purchased from Life Technologies, Inc (Grand Island, NY). The oligonucleotides for PCR amplification (see below) were purchased from Bio-Synthesis Inc. (Lewisville, TX). The RNA oligo was purchased from IDT Inc. (Coralville, IA). AmpliTaq DNA polymerase and deoxynucleotides were obtained from Perkin Elmer/Cetus (Norwalk, CT). The restriction endonucleases and the DNA modification enzymes were purchased from either Bethesda Research Laboratories (Bethesda, MD) or New England Biolabs (Beverley, MA). The T_7_, T_3_, and SP-6 RNA polymerase reagent kits for synthesis of labelled and unlabelled RNAs were purchased from Promega (Madison, WI). CD437, CHX and ActD were from Sigma Chemical Co. (St. Louis, MO).

### Cell lines and Cell Culture

The MDA-MB-468 and MCF-7 HBC cells, and HL-60R human leukemia cells have been described [8,12,13]. HBC cells were grown in Dulbecco's Modified Eagles Medium/Ham's F-12 medium supplemented with 5% FBS, 20 mM Hepes, and 50 μg/ml gentamicin. HL-60 leukemia cells were grown in RPMI 1640 medium supplemented with 5% heat-inactivated FBS and 25 μg/ml gentamicin.

### Cloning of Plasmid Constructs

The construction of the plasmid encoding CMV promoter-driven rabbit β-globin (RBG) mRNA has been described [12]. Plasmids expressing various rabbit β-globin-p21^WAF1/CIP1 ^(WAF) chimeric transcripts were generated as follows. First, an ~1.0 kb fragment of the p21^WAF1/CIP1 ^3'-UTR spanning positions 1006-2004 [1] present in the plasmid clone 8.7 [13] was isolated by ApaI and StuI digestions, end-filled by Klenow DNA polymerase and gel purified. This fragment was ligated in the sense orientation in the above plasmid encoding CMV promoter-driven RBG mRNA that was BglII-digested and blunt-ended to obtain a recombinant plasmid for expression of the RBG-WAF (1006-2004) transcript. Next, the ~1.0 kb insert of plasmid RBG-WAF (1006-2004) was replaced with the sense orientation of XhoI-cut 1.4 Kb subfragment of the p21^WAF1/CIP1 ^3'-UTR (positions 581-2004, [1]) to obtain plasmid for expression of RBG-WAF (581-2004) transcript. Various deletion fragments of the p21^WAF1/CIP1 ^3'-UTR (table 1) were generated by PCR and cloned at the 3' end of rabbit β-globin gene to obtain plasmids encoding RBG-WAF (1498-2004), RBG-WAF (1645-2004), RBG-WAF (1795-2004), RBG-WAF (1900-2004), RBG-WAF (1498-1645), RBG-WAF (1540-1700), RBG-WAF (581-1498), RBG-WAF (581-1195) and RBG-WAF (581-1011) using previously described methodologies [11,12]. In addition, the 0.16 kb subfragment of the p21^WAF1/CIP1 ^3'-UTR present in plasmid RBG-WAF (1540-1700) was subcloned in the sense orientation at the unique HindIII site at the 5' end of the RBG gene to obtain the plasmid for expression of the WAF (1540-1700)-RBG transcript.

The different subfragments of the p21^WAF1/CIP1 ^3'-UTR as well as the GADD45 5'-UTR were also subcloned in the vector plasmid pBluescript KSII (Stratagene) for *in vitro *transcription purposes (table 2). The plasmids pBKS-WAF (581-2110) and pBKS-GADD (10-55) have been described [12,13]. The p21^WAF1/CIP1 ^3'-UTR insert of plasmid RBG-WAF (1540-1700) was subcloned into the pBKSII vector plasmid to obtain the pBKS-WAF (1540-1700). In addition, overlapping subfragments of the p21^WAF1/CIP1 ^3'-UTR region from 1540-1700 were generated by synthesizing 49-mer sense and antisense oligos having 4 nt overhangs for XhoI and XbaI, respectively. Phosphorylation of each of the oligos was carried out by using T_4 _polynucleotide kinase, followed by annealing of the sense oligo with its corresponding antisense partner. The double-stranded, subfragments were independently ligated into pBSK vector to obtain plasmids pBKS-WAF (1540-1585), pBKS-WAF (1575-1620), pBKS-WAF (1610-1655) and pBKS-WAF (1645-1690). All of the recombinant plasmids were sequenced to confirm the orientation and sequences of the inserts.

### RNA Isolation, Northern Blot Analysis and Stable Transfections

The plasmids RBG-WAF (581-2004), RBG-WAF (1006-2004), RBG-WAF (1498-2004), RBG-WAF (1645-2004), RBG-WAF (1795-2004), RBG-WAF (1900-2004), RBG-WAF (1498-1645), RBG-WAF (1540-1700), RBG-WAF (581-1498), RBG-WAF (581-1195), RBG-WAF (581-1011) and WAF (1540-1700)-RBG were independently transfected into MDA-MB-468 HBC cells, followed by selection of several hygromycin (400 μg/ml)-resistant independent sublines from each of the transfection. Total RNA from each stable subline was isolated and expression of respective chimeric transcripts analyzed by the northern blot hybridization using the radiolabelled, NcoI-BamHI digested, gel-purified exon 2 subfragment of the RBG gene as described [12,13]. The northern blot filters were subsequently hybridized with the radiolabelled cDNA fragment corresponding to ribosomal phosphoprotein 36B4 [18] to ascertain RNA loading.

### Analysis of mRNA Decay

Two or more independent hygromycin-resistant MDA-MB-468 sublines with low-to-moderate levels of RBG or RBG-WAF (581-2004) transcripts were selected for analysis of mRNA decay. To study the rate of decay of the RBG or RBG-WAF (581-2004) transcript, CD437-treated or untreated sublines were cultured in the presence of the transcriptional inhibitor ActD (4 μg/ml) for various times followed by isolation of total RNAs and northern blot hybridization as described [12]. For measurement of mRNA decay rates, the data from autoradiograms were quantitated by densitometry using the UN-SCAN-IT software program (Silk Scientific, Inc., Orem, UT). For each sample, the concentration of RBG transcripts was normalized to the signal derived from hybridization with the ribosomal phosphoprotein 36B4 probe. The half-lives of the RBG and RBG-WAF (581-2004) mRNAs were determined essentially as we have described [12].

### In Vitro Transcription and Electrophoretic Gel-mobility Shift Assays

Plasmids pBKS-WAF (581-2110) and pBKS-GADD (10-55) were linearized with NotI and HindIII, respectively, and the T_3 _promoter primer was used to synthesize unlabelled sense strand RNAs as per described methods [19]. In addition, plasmids pBKS-WAF (1540-1585), pBKS-WAF (1575-1620), pBKS-WAF (1610-1655) and pBKS-WAF (1645-1690) were independently linearized with XbaI, and the unlabelled sense strand RNAs were synthesized utilizing T_7 _promoter primer as described [19]. Radiolabelled probes were also prepared from the above linearized plasmid templates to the specific activity of ~10^7 ^cpm/μg of RNA using either [α-^32^P]UTP (3000 Ci/mmol; 10 mCi/ml; NEN), [α-^32^P]CTP (3000 Ci/mmol; 10 mCi/ml; NEN) or a combination of both. The 12 nt RNA oligonucleotide (5'-UGUGGUGGCACA-3') was labelled using T_4 _polynucleotide kinase and [γ^32^P]ATP (3000 Ci/mmol; 10 mCi/ml; NEN) as described [20]. The radiolabelled RNA probes were subsequently purified using G25 spin columns (BMB) or Microcon YM-3 spin cartridges with a 3000-dalton molecular weight cut-off (Millipore, Bedford, MA) and utilized in the gel-mobility shift assays below.

HBC and leukemia cell cytoplasmic as well as nuclear protein extracts were prepared according to procedures described before [20]. HBC cell cytoplasmic extracts were obtained after treatment of cells with CD437 for 0, 3, 6, 12, and 24 hours. The cytoplasmic protein extracts from HL-60R leukemia cells were obtained after treatment with CD437 for 0 and 1 hour. In certain cases, the HBC cells were pretreated with protein synthesis inhibitor CHX (200 μg/ml) or transcriptional inhibitor ActD (4 μg/ml) for 1-2 hours prior to treatment with CD437. The protein extracts (20 μg protein per reaction) were preincubated at room temperature for 10 minutes with 200 ng/μl E. coli tRNA, and 1× binding buffer containing 12 mM HEPES pH7.5, 5 mM MgCl_2_, 1.25 mM EDTA, 1.25 mM DTT, 155 mM KCl and 10% glycerol in a total reaction volume of 9 μl. In some cases, proteinase K (Sigma; 5 μg/reaction), calf intestinal alkaline phosphatase (Boeringer Mannheim Biochemicals; 40 units/reaction), or 2 μl of ~100 ng/μl unlabelled sense strand RNAs were included in the pre-incubation step. The specific competitor RNAs consisted either a160 nt long RNA derived from plasmid pBKS-WAF (1540-1700) or an ~1500 nt RNA derived from plasmid pBKS-WAF (581-2110). The nonspecific competitor RNA consisted of an ~400 nt 3'-UTR subfragment of c-myc mRNA containing an additional 100 nt poly A sequences derived from in vitro transcription of HindIII-digested plasmid pMycSD3, essentially as described [21]. Radiolabelled probe RNA (1 ng/rxn ~10,000 cpm) was then added to the reaction mix and allowed to incubate at room temperature for additional 30 minutes. Reactions were then exposed to UV on ice for 5 min (120 μjoule/min) using UV-crosslinker (Stratagene) to cross-link the proteins interacting with the RNAs. Five units of RNAse T1 (BMB) and 2.5 units of RNAse A (Sigma) were added to each reaction and incubated at room temperature for 30 minutes. Fifty μg of heparin (Sigma) was added to each reaction, followed by incubation on ice for 10 minutes. The reactions were boiled and then analyzed on 12% SDS-polyacrylamide gel (acrylamide:bisacrylamide, 30:0.8) at 65 V in 1× Tris glycine buffer for a period of 14 to 16 h. Gels were dried and exposed for autoradiography for appropriate periods of time.

## Abbreviations

CD437: (6-[3-adamantyl-4-hydroxyphenyl]-2-naphthalene); HBC: Human Breast Carcinoma; CDKI: Cyclin-dependent Kinase Inhibitor; ActD: Actinomycin D; CHX: Cycloheximide; ER: Estrogen Receptor; UTR: Untranslated region; FBS: Fetal bovine serum; PCR: Polymerase chain reaction; nt: nucleotides; bp: base pairs; Kb: kilobase pairs; kD: kiloDalton.

## Competing interests

The authors declare that they have no competing interests.

## Authors' contributions

LZ and AKR carried out the majority of experiments including cloning of chimeric RBG constructs, RNA preparations, half-life determinations, northern blots, and analysis of RNA-protein interactions. AW, MID and JAF participated in the design and discussion of various experiments. AKR is the corresponding author responsible for experimental design and coordination, as well as writing of the manuscript and formatting of all the figures. All authors read and approved the final manuscript.
